# Association of Adherence Support and Outreach Services with Total Attrition, Loss to Follow-Up, and Death among ART Patients in Sub-Saharan Africa

**DOI:** 10.1371/journal.pone.0038443

**Published:** 2012-06-07

**Authors:** Matthew R. Lamb, Wafaa M. El-Sadr, Elvin Geng, Denis Nash

**Affiliations:** 1 International Center for AIDS Care and Treatment Programs (ICAP), Mailman School of Public Health, Columbia University, New York, New York, United States of America; 2 Department of Epidemiology, Mailman School of Public Health, Columbia University, New York, New York, United States of America; 3 College of Physicians and Surgeons, Columbia University, New York, New York, United States of America; 4 Division of HIV/AIDS at San Francisco General Hospital, University of California San Francisco, San Francisco, California, United States of America; 5 CUNY School of Public Health at Hunter College, New York, New York, United States of America; Boston University, United States of America

## Abstract

**Background:**

Loss to follow-up (LTF) after antiretroviral therapy (ART) initiation is common in HIV clinics. We examined the effect of availability of adherence support and active patient outreach services on patient attrition following ART initiation.

**Methods and Findings:**

This ecologic study examined clinic attrition rates (total attrition, LTF, and death) among 232,389 patients initiating ART at 349 clinics during 2004–2008 in 10 sub-Saharan African countries, and cohort attrition (proportion retained at 6 and 12 months after ART initiation) among a subset of patients with follow-up information (n = 83,389). Log-linear regression compared mean rates of attrition, LTF, and death between clinics with and without adherence support and outreach services. Cumulative attrition, LTF, and death rates were 14.2, 9.2, and 4.9 per 100 person-years on ART, respectively. In multivariate analyses, clinic availability of >2 adherence support services was marginally associated with lower attrition rates (RR_adj_ = 0.59, 95%CI: 0.35–1.0). Clinics with availability of counseling services (RR_adj_ = 0.62, 95%CI: 0.42–0.92), educational materials (RR_adj_ = 0.73, 95%CI: 0.63–0.85), reminder tools (RR_adj_ = 0.79, 95%CI: 0.64–0.97), and food rations (RR_adj_ = 0.72, 95%CI: 0.58–0.90) had significantly lower attrition, with similar results observed for LTF. Outreach services were not significantly associated with attrition. In cohort analyses, attrition was significantly lower at clinics offering >2 adherence support services (RR_adj,6m_ = 0.84, 95%CI: 0.73–0.96), dedicated pharmacy services (RR_adj,6m_ = 0.78, 95%CI: 0.69–0.90), and active patient outreach (RR_adj,6m_ = 0.85, 95%CI: 0.73–0.99). Availability of food rations was marginally associated with increased retention at 6 (RR_adj,6m_  = 0.82, 95%CI: 0.64–1.05) but not 12 months (RR_adj,12m_  = 0.98, 95%CI: 0.78–1.21).

**Conclusions:**

Availability of adherence support services, active patient outreach and food rations at HIV care clinics may improve retention following ART initiation.

## Introduction

Adherence to antiretroviral therapy (ART) and long-term retention in care is essential for optimal treatment outcomes. Identifying modifiable clinic-level factors associated with patient retention and survival may suggest feasible points of intervention.

Two reviews of patients initiated on ART in sub-Saharan Africa reported high non-retention six (12%–45%) and 12 (10%–51%) months after ART initiation with substantial variability across clinics [1], [2]. Studies tracing patients lost to follow-up (LTF) have found high unascertained deaths and transfers [3]–[5], suggesting both contribute substantially to LTF.

Services focusing on barriers to medication and care adherence, including forgetfulness [6]–[10], lack of knowledge about the importance of adherence [6]–[12], fear of increased appetite coupled with food insecurity[10], [12]–[14], and stigma [10]–[13] may improve retention by increasing survival and reducing LTF, but their effectiveness in a diverse service delivery context is largely unknown. Two studies in resource-limited settings have found active outreach associated with lower LTF and more complete vital status ascertainment [15], [16].

This study used routinely-collected aggregate (ecologic) data collected from HIV care and treatment clinics in 10 sub-Saharan African countries to investigate whether the availability of clinic services targeting adherence to ART medication and retention in care was associated with better retention after ART initiation.

## Methods

### Study Population

All HIV care and treatment programs supported by the U.S. President’s Emergency Plan for AIDS Relief (PEPFAR) are required to report to the U.S. Government aggregate data summarizing characteristics of patients receiving HIV care and treatment at these clinics. Our study used aggregate (ecologic) clinic-level information from HIV-positive patients receiving antiretroviral therapy at clinics supported by ICAP-Columbia University in 10 sub-Saharan African countries (Cote d’Ivoire, Ethiopia, Kenya, Lesotho, Mozambique, Nigeria, Rwanda, South Africa, Tanzania, Zambia) were included. Each clinic is governed by national HIV care guidelines and provides free ART. Information on clinic characteristics was collected for routine monitoring and evaluation purposes by ICAP-staff through interviews conducted with clinic staff. No patient-level information was used in this study. The use of these routine monitoring and evaluation data for this study was approved as nonhuman subjects research by the US Centers for Disease Control and Prevention (CDC) and the Institutional Review Board of Columbia University Medical Center.

We included clinics providing ART services during January 2004–December 2008 that reported quarterly care and treatment indicators for at least three consecutive quarters and completed a site assessment survey. Of the 392 clinics supported by ICAP during 2004–2008, 349 (89%) were included, representing over 232,000 patients initiating ART. Six and 12 month follow-up data were also available on 1,097 cohorts of patients (N  = 83,389) initiating ART in 3 month quarterly calendar periods, and were included in cohort analyses to assess retention on ART.

### Data Sources

Three sources of data were used: (1) cumulative clinic-level data on the number of patients initiating ART, LTF, and reported as having died or transferred (reported quarterly); (2) cohort-level data on the proportion of persons initiating ART in a quarter who remained on ART six- and 12-months after ART initiation (also reported quarterly), and (3) a structured survey administered to clinic staff measuring clinic-level characteristics.

#### Aggregate clinic-level outcome data

Aggregate clinic-level data were obtained from routinely-collected PEPFAR Track 1.0 quarterly program indicators, manually tallied from registers by clinic staff. Each clinic has a paper-based patient register provided by the country Ministry of Health, on which visit date information is captured. Clinic staff record patients who transfer to another clinic as “transfers out”, and those not seen for more than three months as lost to follow-up on these forms; LTF patients who return to care will have their LTF designation removed from the register. Separate “ART registers” are tabulated by clinic data clerks to retrospectively assess whether patients initiating ART 6 and 12 months ago are alive and on ART during the current quarter.

#### Program-level exposure data

Availability of adherence support and outreach services at each clinic, along with information on the context in which each clinic operates, was obtained from structured surveys administered by field staff in June 2007, December 2007, and July 2008. Test-retest agreement assessed for a subset of survey items at 58 clinics included in this analysis was 83% overall (79% for adherence support questions, 74% for the outreach question) (data not presented).

### Outcome Definitions

Attrition was defined as the sum of patients initiating ART who were reported: 1) dead, 2) LTF, or 3) discontinued ART (even if they remained in HIV care) during the reporting period. LTF was defined as having no clinic visit in 3 months without documented evidence of death or transfer to another clinic. In the cumulative analysis, patients considered LTF as per above definition who return to ART care were no longer considered LTF, while in the cohort analysis such patients were LTF at 6 and 12 months regardless of whether they subsequently returned thereafter to ART care. Patients were classified as having died based on information passively received by each clinic; no deaths were independently ascertained.

#### Cumulative rates (attrition, LTF, death)

**Figure 1:**
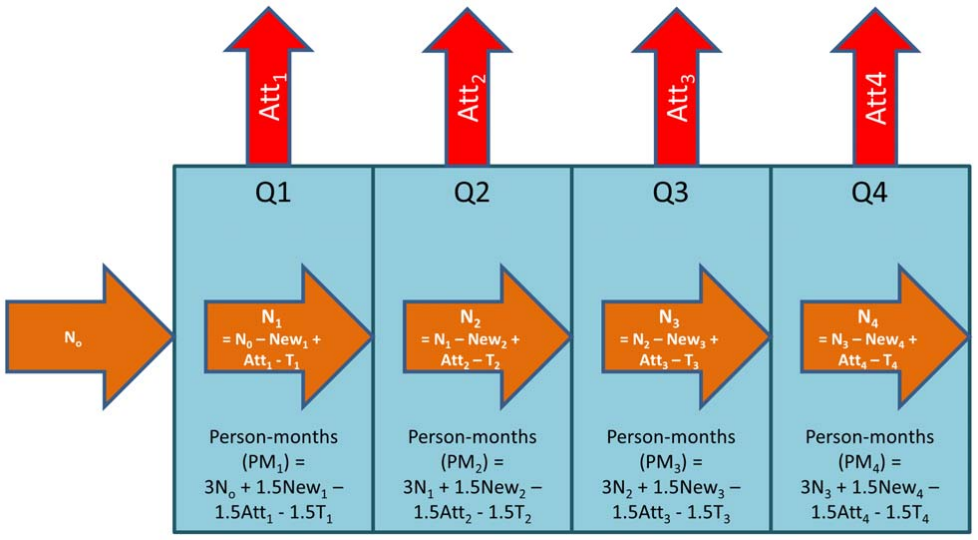
Schematic calculation of clinic-level attrition, LTF, and death rates using aggregate data. N_i_  =  Number of patients active on ART at the end of reporting quarter i. New_i_  =  Number of patients newly initiating ART during reporting quarter i. Att_i_  =  Number of patients attritioned (discontinued ART, lost to follow-up, or dead) during quarter i. T_i_  =  Number of patients transferring to another clinic during quarter i.

Clinic person-time on ART during each quarter was calculated by allotting 3 months of person-time for each patient on ART at the beginning of a given quarter, and 1.5 months for patients initiating ART or discontinuing ART (death, transfer, LTF, stopping ART) during the quarter. Thus, all attrition and ART initiation was assumed to occur at the middle of each quarter. These person-months were summed across all quarters through December 2008 to obtain cumulative clinic person-time on ART. Total attrition, death, and LTF rates were computed by dividing the cumulative number of attritions, deaths, or LTF, respectively, by the cumulative clinic person-time on ART for each clinic. Rates through December 2008 were expressed per 100 person-years on ART. Figure 1 describes the method used to calculate clinic-level attrition, LTF, and death rates using the example of total attrition.

For example, if 100 patients were active on ART at the beginning of the study period (N_0_ in Figure 1, 25, 30, 35, and 30 patients newly initiated ART in Quarters 1, 2, 3, and 4, respectively, and the total attrition was 5, 10, 15, and 10 patients during Quarters 1, 2, 3, and 4, respectively, while there were no transfers during this period, our cumulative attrition rate for this clinic is calculated as:

Total attrition  =  Sum(Att_1_ to Att_4_)  = 5+10+15+10 = 40 patients

PM_1_ = 100patients*3months + 25patients*1.5months – 5patients*1.5months  = 330 person- months

PM_2_ = 120patients*3months + 30patients*1.5months – 10patients*1.5months  = 390 person-months

PM_3_ = 140patients*3months + 35patients*1.5months – 15patients*1.5months  = 450 person-months

PM_4_ = 160patients*3months + 30patients*1.5months – 10patients*1.5months  = 510 person-months

Cumulative attrition rate  = 40/(330+390+450+510)  = 28.6 per 100 person-years

#### Cohort attrition 6 and 12 months following ART initiation

Data on 6 and 12 month attrition was derived from a subset of patients (83,389 of the 232,000 total patients) from 1,097 three-month cohorts of patients initiating ART. Cohorts were included if data were available at both 6 and 12 months after ART initiation, and cohort attrition was defined as the proportion of patients not alive and on ART 6 and 12 months after ART initiation at a given clinic.

### Clinic Adherence and Outreach Services

Annual structured site assessments conducted at each clinic captured information on clinic staffing and the availability of support services. For this analysis, we focused on support services targeting adherence to antiretroviral therapy and retention in care. Clinics were queried on availability of the following categories of adherence support services: “directed support services” involving interaction with clinic staff (one-on-one or group adherence counseling, on-site support groups, peer educator programs), “informational services” (written educational materials, reminder tools), “pharmacy services” (routine medication pickup review, presence of a dedicated ART pharmacist), and “structural services” (food rations to support ART adherence). Each service was dichotomized according to reported availability at clinic. Additionally, the total number of adherence support services available at a given clinic (dichotomized at the lower quintile of prevalence into >2 vs. ≤2) was constructed. Clinics reporting the presence of a system to trace patients missing scheduled visits via telephone, letters, or home visits were classified as having active outreach.

### Statistical Analyses

Cumulative clinic rates of total attrition, LTF, and death, as well as 6 and 12 month attrition proportions for cohorts initiating ART, were assessed in relation to clinic availability of adherence and outreach services. Adjustments were made for clinic characteristics thought to be plausibly associated with patient outcomes (clinic population (cumulative number of patients enrolled in care), location (urban/rural), facility type (primary, secondary, tertiary), year of HIV program start, and year of ART initiation (for cohort analyses)).

Log-linear models were used to estimate cumulative attrition, LTF, and death rate ratios comparing clinics with and without availability of each adherence support and outreach service. Both unadjusted and adjusted models controlling for clinic characteristics listed above were fit. Next, a “full” model was constructed to assess the joint influence of each adherence and active outreach service associated with the outcome of interest at alpha level ≤0.1.

For 6 and 12 months cohort attrition, risk ratios comparing mean attrition proportions of cohorts at 6 and 12 months after ART initiation were modeled using log-linear regression accounting for within-site correlation following methods described above.

A series of sensitivity analyses were conducted on our cumulative and ART cohort analyses excluding years of data collection and individual countries to estimate the influence on our measures of association. These sensitivity analyses follow the same procedures outlined above.

## Results

232,000 patients initiated ART from 349 clinics in 10 countries and were followed for 300,700 person-years. Most clinics were situated at primary (47%) or secondary (48%) health facilities, and 57% were located in semi-urban or urban areas (Table 1). Kenya contributed the most number of clinics (N = 71 [20%]), while Mozambique contributed the most number of patients (N = 53,000 [23%]). Overall, 59% of the adults were female and 8% were pediatric patients <15 years of age.

**Table 1:** Facility and cohort-level characteristics of study population.

	Facility-Level Characteristics				12-month Cohort-level Characteristics						
	N (%) Facilities		Cum N (%) patients on ART		N (%) facilities with cohorts		N (%) cohorts		N (%) patients in cohorts		median (q1–q3) patients per cohort per facility
**Total**	349	(100%)	232,389	(100%)	221	(63%)	1,097	(100%)	83,389	(100%)	48 (21–96)
Adult			213,693	(92%)							
Adult Female			136,999	(59%)	Median CD4 count at ART initiation:				134 (109–171)		
Pediatric			18,696	(8%)							
**General Facility Characteristics**											
Cote d’Ivoire	9	(2.6%)	980	(0.4%)							
Ethiopia	44	(12.6%)	37,374	(16.1%)	38	(17.2%)	244	(22.2%)	20,212	(24.2%)	49 (24–116)
Kenya	71	(20.3%)	26,001	(11.2%)	33	(14.9%)	113	(10.3%)	6,410	(7.7%)	44 (18–80)
Lesotho	26	(7.4%)	18,117	(7.8%)	11	(5.0%)	36	(3.3%)	4,362	(5.2%)	103 (52–178)
Mozambique	39	(11.2%)	53,315	(22.9%)	36	(16.3%)	196	(17.9%)	23,023	(27.6%)	72 (28–186)
Nigeria	27	(7.7%)	19,478	(8.4%)	12	(5.4%)	36	(3.3%)	5,873	(7%)	124 (88–181)
Rwanda	44	(12.6%)	19,755	(8.5%)	39	(17.7%)	240	(21.9%)	8,334	(10%)	27 (11–43)
South Africa	43	(12.3%)	37,620	(16.2%)	31	(14.0%)	148	(13.5%)	8,961	(10.7%)	47 (21–84)
Tanzania	45	(12.9%)	19,202	(8.3%)	21	(9.5%)	84	(7.7%)	6,217	(7.5%)	56 (32–91)
Zambia	1	(0.3%)	547	(0.2%)	.	.	.		.		.
Facility Type											
Primary	163	(46.7%)	49,434	(21.3%)	85	(38.5%)	354	(32.3%)	14,829	(17.8%)	24 (13–50)
Secondary	168	(48.1%)	160,020	(68.9%)	122	(55.2%)	646	(58.9%)	56,978	(68.3%)	60 (31–115)
Tertiary	14	(4.0%)	22,848	(9.8%)	12	(5.4%)	88	(8%)	10,842	(13%)	113 (46–192)
Facility Location											
Rural	149	(42.7%)	30,484	(13.1%)	79	(36.0%)	341	(31.1%)	9,850	(11.8%)	24 (15–36)
Semi-urban	114	(32.7%)	90,409	(38.9%)	75	(34.0%)	333	(30.4%)	23,519	(28.2%)	52 (25–96)
Urban	80	(22.9%)	111,427	(47.9%)	65	(29.0%)	410	(37.4%)	49,228	(59%)	84 (49–162)
Year site initiated ART care (site-level), or year cohort initiated ART (cohort-level)											
2003	7	(2%)	14,583	(6.3%)			–		–		–
2004	41	(11.7%)	76,924	(33.1%)			–		–		–
2005	73	(20.9%)	72,391	(31.2%)	n.a		6	(0.5%)	132	(0.2%)	25 (10–32)
2006	74	(21.2%)	39,457	(17%)	n.a		58	(5.3%)	4,747	(5.7%)	61 (31–100)
2007	59	(16.9%)	20,042	(8.6%)	n.a		333	(30.4%)	26,145	(31.4%)	48 (20–105)
2008	94	(26.9%)	8,992	(3.9%)	n.a		700	(63.8%)	52,366	(62.8%)	48 (21–94)

### Adherence Support and Outreach Services

Almost all clinics (93%) reported availability of at least one adherence support service and 83% reported more than two; 53% reported availability of active patient outreach (Table 2). Clinics averaged availability of four adherence support services; specific service availability at clinics ranged from 17% (food rations) to 88% (one-on-one and/or group counseling). Table 3 presents cross-tabulated proportions showing the joint distribution of adherence support and outreach services within the same facilities. We found that adherence support services were not randomly distributed across clinics. Rather, clinics with one service tended to be more likely to have other services as well, although there is substantial heterogeneity between clinics.

**Table 2:** Facility and cohort-level exposure and outcome characteristics.

		Facility-Level Characteristics				Cohort-level Characteristics (N = 221 facilities with cohorts)				
		N (%) of facilities		Cum N (%) patients on ART		Number (%) of cohorts		Number (%) of patients in cohorts		median (q1–q3) patients per cohort per site
**Overall**		349	(100%)	232,389	(100%)	1,097	(100%)	83,389	(100%)	48 (21–96)
**Adherence support and related services**										
Any adherence support program	no	15	(4.3%)	465 396	(0.2%)	1	(0.1%)	10	(0%)	10 (10–10)
	yes	326	(93.4%)	231,924 231,783	(99.7%)	1082	(98.6%)	82611	(99.1%)	48 (22–96)
**Directed support services**										
one-on-one/group adherence counseling	no	41	(11.7%)	7,532	(3.2%)	403	(36.7%)	30,947	(37.1%)	48 (21–99)
	yes	308	(88.3%)	224,857	(96.8%)	685	(62.4%)	51,701	(62%)	48 (21–94)
on-site support groupsfor HIV+ patients	no	159	(45.6%)	62,447	(26.9%)	409	(37.3%)	24,238	(29.1%)	38 (18–74)
	yes	190	(54.4%)	169,942	(73.1%)	679	(61.9%)	58,411	(70%)	59 (25–115)
peer educator program	no	199	(57%)	105,070	(45.2%)	612	(55.8%)	37,115	(44.5%)	39 (19–80)
	yes	150	(43%)	127,319	(54.8%)	476	(43.4%)	45,534	(54.6%)	61 (26–128)
**Informational services**										
Educational materials promoting ART adherence	no	150	(43%)	85,929	(37%)	315	(28.7%)	26,578	(31.9%)	49 (18–110)
	yes	199	(57%)	146,460	(63%)	773	(70.5%)	56,070	(67.2%)	47 (23–94)
Reminder tools (e.g., clocks, calendars, pill boxes)	no	104	(29.8%)	27,522	(11.8%)	110	(10%)	7,844	(9.4%)	33 (17–82)
	yes	245	(70.2%)	204,867	(88.2%)	978	(89.2%)	74,805	(89.7%)	49 (22–99)
**Pharmacy Services**										
Routine medication pickup review, dedicated or team pharmacist	no	71	(20.3%)	9,253	(4%)	61	(5.6%)	3,220	(3.9%)	24 (12–55)
	yes	278	(79.7%)	223,136	(96%)	1,027	(93.6%)	79,428	(95.3%)	49 (23–99)
**Structural services**										
Food rations to promote ART adherence	no	289	(82.8%)	198,231	(85.3%)	919	(83.8%)	76,078	(91.2%)	53 (23–109)
	yes	60	(17.2%)	34,158	(14.7%)	178	(16.2%)	7,311	(8.8%)	27 (17–57)
**Outreach Services**										
Active patient outreach	no	164	(47%)	63,969	(27.5%)	363	(33.1%)	25,037	(30%)	44 (21–83)
	yes	185	(53%)	168,420	(72.5%)	725	(66.1%)	57,611	(69.1%)	50 (22–105)

**Table 3:** Cross-tabulated frequency of adherence support and outreach services.

		Adherence counseling		On-site support groups for HIV+ patients		Peer educators		Educational materials		Reminder tools		Routine medication pickup review/dedicated pharmacist		Food rations to promote ART adherence		Active patient outreach	
		Yes	No	Yes	No	Yes	No	Yes	No	Yes	No	Yes	No	Yes	No	Yes	No
Adherence counseling	Yes			57%	43%	45%	55%	62%	37%	77%	23%	87%	13%	19%	81%	58%	42%
	No			37%	63%	73%	27%	20%	80%	20%	80%	22%	78%	0%	100%	15%	85%
On-site support groupsfor HIV+ patients	Yes	92%	8%			64%	36%	61%	39%	79%	21%	85%	15%	24%	76%	78%	22%
	No	84%	16%			18%	82%	52%	47%	60%	40%	74%	26%	9%	91%	23%	77%
Peer educators	Yes	93%	7%	81%	19%			64%	36%	80%	20%	85%	15%	29%	71%	81%	19%
	No	85%	15%	34%	66%			52%	48%	63%	37%	76%	24%	8%	92%	32%	68%
Educational materials	Yes	96%	4%	58%	42%	48%	52%			86%	14%	94%	6%	28%	72%	62%	38%
	No	78%	22%	50%	50%	36%	64%			49%	51%	60%	40%	3%	97%	41%	59%
Reminder tools	Yes	97%	3%	61%	39%	49%	51%	70%	30%			92%	8%	21%	79%	63%	37%
	No	68%	32%	38%	62%	29%	71%	26%	74%			50%	50%	8%	92%	30%	70%
Routine medication pickup review/dedicated pharmacist	Yes	97%	3%	58%	42%	46%	54%	68%	32%	81%	19%			21%	79%	60%	40%
	No	55%	45%	41%	59%	32%	68%	15%	85%	27%	73%			1%	99%	27%	73%
Food rations to promote ART adherence	Yes	100%	0%	75%	25%	73%	27%	92%	8%	87%	13%	98%	2%			98%	2%
	No	86%	11%	50%	50%	37%	63%	50%	50%	67%	33%	76%	24%			44%	56%
Active patient outreach	Yes	97%	3%	80%	20%	65%	35%	66%	34%	83%	17%	89%	11%	32%	68%		
	No	79%	21%	26%	74%	18%	82%	46%	54%	55%	45%	68%	32%	1%	99%		

### Outcomes

Of the 232,389 patients initiating ART during 2004- December 2008, 72% were still active on ART and attending the same clinic at the end of the follow-up period. Of those no longer active on ART as of December 2008, 20,348 (9%) transferred to another clinic and attrition occurred among 44,428 (19%), including 14,678 (6.3%) who died, 2,148 (0.9%) discontinuing ART, and 27,602 (11.9%) LTF. The overall total attrition rate (measured as specified in Figure 1) was 14.2 per 100 person-years (4.9 deaths, 0.7 discontinuations, and 9.2 LTF per 100 person-years). Among the 6 and 12 month ART cohorts, attrition proportion were 20% and 27%, respectively among the 82,981 patients included.

### Total Attrition

In multivariate analyses (Table 4), clinics with >2 vs. ≤2 adherence support services available had marginally lower attrition (RR_adj_ = 0.59, 95%CI: 0.35–1.0). For specific adherence services, availability of educational materials (RR_adj_ = 0.73, 95%CI: 0.63–0.85), one-on-one and/or group adherence counseling (RR_adj_ = 0.62, 95%CI: 0.42–0.92), reminder tools (RR_adj_ = 0.79, 95%CI: 0.64–0.97), and food rations to support ART adherence (RR_adj_ = 0.72, 95%CI: 0.58–0.90) were significantly associated with lower attrition rates (Table 4). On-site support groups, peer educator programs, pharmacy support services and active patient outreach were not significantly associated with attrition following ART initiation.

**Table 4:** Crude and Adjusted overall Rate Ratios for attrition, loss to follow-up, and death.

Facility-Level Characteristics				Overall Attrition^1^ Rate Ratio				Overall Loss to Follow-up^2^ Rate Ratio										Overall Death^3^ Rate Ratio										
			N (yes/no)	Crude(95%CI)		Adjusted^4^(95%CI)		Crude(95%CI)					Adjusted^4^(95%CI)					Crude(95%CI)				Adjusted^4^ (95%CI)						
**Adherence support services**																												
Total number of adherence support services provided	>2 vs. ≤2		292/57	0.51	(0.31–0.85)	0.59	(0.35–1.0)	0.45		(0.24–0.84)		0.48			(0.25–0.92)		0.7			(0.38–1.28)			0.94		(0.55–1.61)			
**Directed support services**																												
One-on-one/group adherence counseling services	yes vs. no		308/41	0.58	(0.4–0.86)	0.62	(0.42–0.92)	0.52	(0.32–0.84)		0.55			(0.33–0.89)		0.75			(0.48–1.17)		0.82			(0.55–1.21)				
on-site support groups for HIV+patients	yes vs. no		190/159	1.06	(0.89–1.25)	1.03	(0.87–1.22)	1.24	(0.99–1.55)		1.20			(0.95–1.52)		0.80			(0.69–0.94)		0.81			(0.7–0.93)				
Peer educator program	yes vs. no		150/199	1.10	(0.95–1.27)	0.99	(0.86–1.14)	1.16	(0.96–1.4)		1.08			(0.89–1.32)		0.98			(0.85–1.14)		0.84			(0.74–0.96)				
**Informational services**																												
Educational materials promoting ART adherence	yes vs. no		199/150	0.83	(0.72–0.96)	0.73	(0.63–0.85)	0.69	(0.57–0.83)		0.63			(0.52–0.77)		1.19			(1.02–1.4)		0.98			(0.85–1.13)				
Reminder tools (e.g., clocks, calendars, pill boxes)	yes vs. no		245/104	0.79	(0.64–0.98)	0.79	(0.64–0.97)	0.79	(0.6–1.05)		0.77			(0.58–1.02)		0.79			(0.63–0.99)		0.81			(0.66–0.98)				
**Pharmacy services**																												
Routine medication pickup review, dedicated or team pharmacist	yes vs. no		278/71	0.61	(0.42–0.91)	0.71	(0.48–1.05)	0.59	(0.36–0.96)		0.60			(0.36–1)		0.68			(0.45–1.03)		0.95			(0.66–1.37)				
**Structural services**																												
Food rations to promote ARTadherence	yes vs. no		60/289	0.83	(0.66–1.03)	0.72	(0.58–0.9)	0.66	(0.49–0.9)		0.65			(0.47–0.88)		1.16			(0.95–1.41)		0.83			(0.69–1)				
**Outreach Services**																												
Active patient outreach	yes vs. no		185/164	0.97	(0.82–1.14)	1.00	(0.85–1.18)	1.03	(0.83–1.28)		1.05			(0.84–1.32)		0.87			(0.74–1.02)		0.91			(0.79–1.06)				

1 Overall attrition rates estimated as the cumulative number of patients at a site lost to follow-up, withdrawn, or reported dead, over the total person-years observed on ART at that site.

2 Overall loss to follow-up rates estimated as the cumulative number of patients not returning to clinic for >6 months since last visit, with no known status, over the total person-years observed on ART at that site.

3 Overall death rates estimated as the cumulative number of patients reported dead, over the total person-years observed on ART at that site.

4 Adjusted for facility type (primary, secondary, or tertiary), urban/rural, year facility began providing ART care, and cumulative number of patients seen in care.

### Loss to Follow-up

In multivariate analyses, clinics with >2 vs. ≤2 adherence support services available had lower rates of LTF (RR_adj_  = 0.48, 95%CI: 0.25–0.92). For specific services, availability of educational materials (RR_adj_ = 0.63, 95%CI: 0.52–0.77) and adherence counseling (RR_adj_ = 0.55, 95%CI: 0.33–0.89) was significantly associated with lower rates of LTF. Availability of pharmacy services including routine medication pickup review (RR_adj_ = 0.60, 95%CI: 0.36–1.0) was marginally associated with LTF, as was availability of food rations (RR_adj_ = 0.65, 95%CI: 0.47–0.88). Other service availability was not significantly associated with LTF.

### Deaths

In multivariate analyses (Table 4), availability of >2 vs. ≤2 adherence support services was not associated with reported death rates. Among specific adherence services, availability of on-site support groups (RR_adj_ = 0.81, 95%CI: 0.70–0.93), peer educators (RR_adj_ = 0.84, 95%CI: 0.74–0.96) and reminder tools (RR_adj_ = 0.81, 95%CI: 0.66–0.98) were significantly associated with lower death rates, while availability of food rations to support ART adherence was marginally associated with lower death rates (RR_adj_ = 0.83, 95%CI: 0.69–1.0). Availability of adherence counseling, educational materials, and pharmacy services were not significantly associated with death rates at clinics.

### Attrition in 6 and 12 Month Cohorts

In multivariate analyses of 6 and 12 months cohorts of patients initiating ART (Table 5), availability of >2 vs. ≤2 adherence support services had significantly lower cohort attrition at 6 months (RR_adj_ = 0.84, 95%CI: 0.73–0.96), but not 12 months following ART initiation. Cohorts of patients initiating ART at clinics offering pharmacy services including routine medication pickup review were significantly associated with lower attrition at 6 months and marginally lower attrition at 12 months, respectively (RR_adj,6m_ = 0.78, 95%CI: 0.69–0.90; RR_adj,12m_ = 0.85, 95%CI: 0.73–1.0). Cohorts of patients initiating ART at clinics with active patient outreach had lower attrition at 6 and 12 months (RR_adj,6m_ = 0.86, 95%CI: 0.73–0.99; RR_adj,12m_ = 0.84, 95%CI: 0.74–0.96, respectively). Availability of other services were not significantly associated with 6 or 12 month attrition among cohorts of patients initiating ART.

**Table 5:** Cohort analysis^4^: Crude and Adjusted Risk Ratios for attrition through 6 and 12 months.

Facility-Level Characteristics			Attrition Risk Ratio through 6 months^1^				Attrition Risk Ratio through 12 months^2^					
		N (yes/no)	Crude RR (95%CI)		Adjusted^3^ RR (95%CI)		Crude RR (95%CI)		Adjusted^3^ RR (95%CI)			
**Adherence support services**												
Total number of adherence support services provided	>2 vs. ≤2	1016/81	0.82	(0.71–0.95)	0.84	(0.73–0.96)	0.9	(0.76–1.07)		0.89	(0.75–1.05)	
**Directed support services**												
one–on-one/group adherence counseling	yes vs. no	685/403	1.13	(1.01–1.26)	1.07	(0.96–1.2)	1.28	(1.12–1.45)		1.22	(1.1–1.36)	
on-site support groups for HIV+ patients	yes vs. no	679/409	0.91	(0.72–1.14)	0.90	(0.74–1.1)	0.92	(0.77–1.11)		0.89	(0.77–1.03)	
peer educator program	yes vs. no	476/612	0.93	(0.74–1.17)	0.93	(0.77–1.12)	0.97	(0.8–1.17)		0.94	(0.81–1.1)	
**Informational services**												
Educational materials promoting ART adherence	yes vs. no	773/315	1.01	(0.84–1.21)	0.97	(0.81–1.17)	1.05	(0.91–1.21)		1.02	(0.87–1.19)	
Reminder tools (e.g., clocks, calendars, pill boxes)	yes vs. no	978/110	1.04	(0.67–1.61)	1.03	(0.7–1.51)	1.03	(0.79–1.36)		1.02	(0.8–1.29)	
**Pharmacy services**												
Routine medication pickup review, dedicated or team pharmacist	yes vs. no	1027/61	0.80	(0.68–0.93)	0.78	(0.69–0.9)	0.89	(0.77–1.03)		0.85	(0.73–1)	
**Structural services**												
Food rations to promote ART adherence	yes vs. no	178/919	0.89	(0.7–1.13)	0.82	(0.64–1.05)	1.01	(0.81–1.25)		0.98	(0.78–1.21)	
**Outreach Services**												
Active patient outreach	yes vs. no	725/363	0.83	(0.71–0.96)	0.85	(0.73–0.99)	0.81	(0.71–0.91)		0.84	(0.74–0.96)	

1 Cohort attrition % estimated as 100 - (number of patients on ART through 6 months/number starting cohort at baseline).

2 Cohort attrition % estimated as 100 - (number of patients on ART through 12 months/number starting cohort at baseline).

3 Adjusted for facility type (primary, secondary, or tertiary), urban/rural, cohort start year, and cumulative number of patients seen in care.

4 All analyses adjusting for within-site correlation using generalized estimating equations.

### Adjustment for Multiple Adherence Support Services

In the “full” model including all services significant at an alpha level of 0.1 (Table 6), availability of educational materials and food rations remained significantly associated with lower rates of total attrition and LTF, while availability of on-site support groups and reminder tools remained significantly associated with lower death rates.

**Table 6:** “Full” model analysis: Risk and Rate Ratios for overall attrition, LTF and death adjusting for other adherence support activities^1^.

Adherence support services		Attrition Rate Ratio^2^	LTF Rate Ratio^2^	Death Rate Ratio^2^
one-on-one/group adherence counseling	yes vs. no	0.77 (0.52–1.14)	0.72 (0.44–1.19)	not in model
on-site support groups for HIV+ patients	yes vs. no	not in model	not in model	0.82 (0.69–0.99)
peer educator program	yes vs. no	not in model	not in model	0.89 (0.76–1.05)
Educational materials promoting ART adherence	yes vs. no	0.76 (0.66–0.89)	0.67 (0.55–0.81)	not in model
Reminder tools (e.g., clocks, calendars, pill boxes)	yes vs. no	0.83 (0.67–1.03)	0.83 (0.63–1.11)	0.78 (0.64–0.94)
Routine medication pickup review, dedicatedor team pharmacist	yes vs. no	0.92 (0.62–1.38)	0.85 (0.50–1.42)	not in model
Food rations to promote ART adherence	yes vs. no	0.74 (0.60–0.92)	0.67 (0.49–0.91)	0.86 (0.71–1.04)
Active patient outreach program	yes vs. no	not in model	not in model	1.12 (0.93–1.34)

1 All models adjusted for year of ART initiation, facility type (primary, secondary, tertiary), facility location (urban/rural), and cumulative number of patients enrolled in care.

2 Rate ratios for total attrition, loss to follow-up, and death additionally adjusted for other adherence support and active outreach services listed in the above table.

For the ART cohort analysis, in the “full” model including all adherence support services significant at an alpha level of 0.1 (Table 7), availability of pharmacy services remained significantly associated with lower attrition at 6 months, while availability of active patient outreach remained significantly associated at 12 but not 6 months. Availability of food rations was not associated with 6 or 12 month attrition among cohorts of patients initiating ART.

**Table 7:** ART cohort “full model” analysis: Adjusted1 Attrition Risk Ratio at 6 and 12 months, adjusting for other adherence support activities

Adherence support services		Attrition % through 6 months	Attrition % through 12 months
		RR^1^	RR^1^
Routine medication pickup review, dedicatedor team pharmacist	yes vs. no	0.81 (0.70–0.94)	0.87 (0.73–1.04)
Food rations to promote ART adherence	yes vs. no	0.87 (0.67–1.12)	1.04 (0.83–1.31)
Active patient outreach program	yes vs. no	0.87 (0.75–1.12)	0.84 (0.73–0.96)

1 Percent attrition ratios (RRs) additionally adjusted for other adherence support and active outreach services listed in the above table.

Sensitivity analyses for both clinic- and cohort-level models examining the impact of excluding the first year of data collection, and separately excluding individual countries, were conducted to assess the robustness of our findings. These analyses found no substantial differences in the magnitude of point estimates, although variability increased somewhat due to a reduced sample size (data not shown).

## Discussion

Most studies of adherence support interventions to date have focused on interventions supporting medication adherence among those receiving antiretroviral medications [14], [17]–[24]. The focus of this report aims at addressing the related and upstream issue of retention of patients after initiation of ART, in recognition of the importance of attrition in the context of large scale HIV programs, which remains suboptimal [1], [2], [9], [25]–[27]. Our findings demonstrate that clinics with educational materials and food rations available were significantly associated with lower attrition and lower LTF compared with clinics without these services, while clinics with availability of support groups, peer educators and reminder tools for adherence were associated with lower rates of measured death compared to clinics without these services. In ART cohort analyses, pharmacy support was significantly associated with lower 6 month attrition, and active outreach was associated with lower 12 month attrition. These findings were observed across clinics from diverse settings in sub-Saharan Africa independent of other clinic characteristics (urban/rural, facility type [primary, secondary, tertiary], patient load, and program maturity).

Studies from sub Saharan Africa have attempted to better define the outcomes of patients lost to follow-up. In our analyses, 65% of the observed attrition rate was due to LTF. A 2009 review of studies tracing patients LTF from resource-limited settings estimated that between 33–48% of such patients classified as LTF had actually died, with substantial variability across studies and populations [3]. Other studies have found substantial contributions from both undocumented deaths and undocumented transfers to LTF [4], [5], [26], [28]–[35]. This heterogeneity underscores that there are many, perhaps divergent, reasons why patients become LTF [36]. A key issue is the contribution of unascertained death to losses to follow-up [4]–[6]. If clinics with higher LTF rates are consequentially reporting proportionately fewer “true” deaths, this would mask the relationship between service availability and actual death through differential death reporting. Consequently, in settings experiencing high loss to follow-up, we recommend using the combined outcome of total attrition as the primary measure of patient outcomes when using aggregate data. When using individual-level data, nomogram approaches can be used for correcting mortality rates for loss to follow-up [37], [38].

In our analyses, we noted several discrepancies that may reflect this complex interplay. Clinics with availability of educational materials, one-on-one/group counseling, and reminder tools had significantly lower rates of attrition and LTF, but were not associated with measured death rates. In addition, clinics with availability of support groups and peer educators had significantly lower death rates but did not have lower rates of attrition or LTF. Differentially lower ascertainment of the true number of deaths among clinics offering these services (due to higher LTF in these clinics) may partially explain these findings. Complete ascertainment of causes of LTF is necessary in order to better understand the associations identified in our analyses [39]. Barring this, estimates of the proportion dead among patients LTF, obtained through tracing studies or simulation, would help clarify the complex relationship between LTF and death ascertainment.

Malnutrition and wasting have been associated with unfavorable HIV disease outcomes [14], [40], [41], and food insecurity may adversely affect HIV related outcomes from two perspectives: as a possible reason for non-adherence to ART (fear of hunger and actual hunger coupled with food insecurity [13]) as well as a structural barrier to retention [42], [43] (lost wages from attending clinic). In our analyses, clinics with availability of food rations had significantly lower attrition and LTF rates in the cumulative analysis, but had only a marginal effect on death rates, the latter effect not retained in the full model analysis. It is possible that under-ascertainment of deaths at facilities with higher LTF could mask the true impact of food support services on survival. In the cohort analyses, similar-magnitude but non-significant associations (compared to the cumulative analysis) were observed for attrition at 6 months, but not 12 months. The inconsistent findings may reflect unmeasured confounding (perhaps the 17% of the clinics offering food rations were more likely to offer other [unmeasured] services improving retention) or inconsistency between cumulative and cohort-derived measures of retention.

Availability of services to track patients missing visits has been associated with reduced LTF [14], [44]–[46]. In our analyses, however, clinics with availability of active outreach services did not have lower attrition, LTF or death rates in the cumulative analysis but they did have significantly lower attrition in the 12 month cohort analysis. The reason for this discrepancy may be due to different definitions for these outcomes in the two types of analyses we utilized (cumulative estimates, which carry with them a clinic’s entire history with respect to retention and survival, and cohort-estimates, which provide more time-defined estimates).

For specific adherence support services (adherence counseling services, educational materials, and reminder tools) associations were observed with lower attrition in the cumulative analyses but not in the 6- and 12-month ART cohort analyses. It is important to note that, as highlighted in Figure 1 and above, the cumulative attrition rates are averages over an entire clinic’s reporting history, and more weight is given to quarters in which more patients were active in care. Although this average estimate accurately reflects the average attrition rate for each clinic, it may not accurately represent attrition rates at any one point in time, especially among clinics experiencing rapid scale-up during the time period if this scale up was also coupled with changing attrition. In contrast, cohort attrition proportions estimate 6 and 12 month attrition after ART initiation by following specific groups of patients initiating ART in the same time period. Reporting on longitudinal cohorts of patients initiating ART in a given time period and followed up for 6 and 12 months is inherently a more difficult task to complete, and not all clinics have the capability of doing this, or were able to do this for the full population included in the cumulative analysis. Thus, cohorts that were reported and thus included in this analysis may be more similar in other aspects impacting retention (such as proper reporting systems in place) regardless of availability of these specific services such that associations observed in the cumulative clinic-level analyses can in fact differ from those observed in the cohort analyses.

Our study has a number of strengths. The use of routinely collected programmatic data enabled inclusion of a large number of patients from diverse HIV care and treatment clinics from 10 sub-Saharan African countries, representing approximately 8% of all patients initiating ART in this region and time [47]. The large number of clinics and contexts enabled examination of many clinic-level characteristics. Our findings were robust to sensitivity analyses, including exclusion of individual countries and cohorts that initiated ART in 2004 and 2005. Finally, the use of two different types of outcomes for retention (cumulative and cohort-based measures) allows examination of the findings in the context of the different limitations inherent in each approach.

There are also several limitations to our analyses. This was an ecologic analysis of the impact of programmatic factors on attrition, and therefore cannot adjust for differences in patient characteristics across clinics and cohorts that also impact attrition, such as CD4 count at ART initiation [38], [48]. However, we also note that programmatic factors likely act at least in part through their impact on these individual-level factors. Additionally, our analyses focused on reported presence or absence of various services at clinics, and we are unable to investigate key issues such as intensity and coverage of these services, per-patient service frequency, or quality of the services provided. Also, while our patient population consisted primarily of adults, 8% of our population was pediatric patients, who may have different determinants of attrition, LTF, and death. Due to limitations in the routinely-collected data, we were unable to separate out our aggregate population into adults and children. Also, while the population of patients included in this report was followed between 2004 and 2008, clinic interviews for availability of services were conducted in 2007 and 2008; some clinics with a different service availability profile prior to the initial 2007 assessment may be misclassified for part of the analysis period. Reported deaths could not be confirmed and, as noted above, the lack of complete vital status ascertainment in the context of high rates of LTF, warrants caution in interpretation of associations with death rates. We therefore place greater emphasis on the combined outcome of attrition when assessing programmatic outcomes. For the cumulative clinic-level attrition analyses, we note that data collected over several years, used to calculate an overall average attrition rate for each facility, may be subject to variable quality. Although standardized data quality measures are in place at all clinics, and data are reviewed routinely for errors and inconsistencies, it is possible that the quality of the data, particularly recording of loss to follow-up and mortality, differed within clinics across this time period. We have no information to assess this possibility and this is an important potential source of information bias in our assessments. Finally, we note in Table 3 that the availability of specific adherence support and outreach services is not distributed randomly across all clinics: clinics reporting availability of one service tend to be more likely to offer additional services as well. While we accounted for this by (1) testing whether the presence of more than 2 services was associated with attrition, LTF, and death and (2) reporting on measures of association between specific adherence services and these outcomes adjusting for other service availability (Table 6), we acknowledge that co-linearity between availability of support services limits our ability to separate out the association of specific services.

In summary, acknowledging the important limitations discussed above, our findings provide insights into the association between the availability of various adherence support and outreach activities and retention on ART in HIV programs in sub-Saharan Africa. Further analyses using patient-level information and measures of service utilization and quality would add further to this study. However, a substantial proportion of sub-Saharan HIV clinics do not have electronic patient-level data systems available, and findings from such analyses may be less generalizable to HIV scale-up clinics in the region. Thus broad ecological analyses of service delivery data and analyses that utilize individual-level information should be leveraged to provide complimentary insights. Our analyses demonstrate the utility of routinely-collected aggregate data for informing program evaluation and design, and suggest that availability of adherence support services, active patient outreach, and food rations at HIV care clinics may improve retention following ART initiation.
